# Supplementary material for: Self-repairing interphase reconstructed in each cycle for highly reversible aqueous zinc batteries
Source: Nat Commun. 2022 Sep 12;13:5348. doi: 10.1038/s41467-022-32955-0 (PMC9468148; doi:10.1038/s41467-022-32955-0)
Supplement: Supplementary file 1 — Supplementary information [file 41467_2022_32955_MOESM1_ESM.pdf]

Supplementary Information

Self-repairing interphase reconstructed in each cycle for highly reversible aqueous zinc batteries

Wenyao Zhang[1,2], Muyao Dong[3], Keren Jiang[1], Diling Yang[1], Xuehai Tan[1], Shengli Zhai[1], Renfei Feng[4], Ning Chen[4], Graham King[4], Hao Zhang[1], Hongbo Zeng[1], Hui Li[3], Markus Antonietti[5], Zhi Li[1,*]

1 Department of Chemical and Materials Engineering, University of Alberta, Edmonton, T6G 1H9, Canada

2 Key Laboratory for Soft Chemistry and Functional Materials, Ministry of Education, Nanjing University of Science and Technology, Nanjing, 210094, China

3 Beijing Advanced Innovation Center for Soft Matter Science and Engineering, Beijing University of Chemical Technology, Beijing, 100029, China

4 Canadian Light Source, Saskatoon, S7N 2V3, Canada

5 Colloid Chemistry Department Department, Max Planck Institute for Colloids and Interfaces, Potsdam, 14424, Germany

Contents

Supplementary Figures

Figure S1. Typical structure and TEM images of the as-synthesized C_3N_4QDs ······················ 4

Figure S2. AFM images of the as-synthesized C_3N_4QDs ·· 5

Figure S3. FT-IR spectroscopy and XPS analysis of the as-synthesized C_3N_4QDs ·················· 6

Figure S4. pH value and Zeta potential of various $ZnSO_4$-C_3N_4QDs electrolytes···················· 7

Figure S5. FT-IR spectroscopy of various $ZnSO_4$-C_3N_4QDs electrolytes ···························· 7

Figure S6. The position and projection motion track of Zn^{2+} ions relative to C_3N_4QD ············· 8

Figure S7. LSV profiles of various $ZnSO_4$-C_3N_4QDs electrolytes ···································· 8

Figure S8. Calculated total DOS of Zn adsorbed C_3N_4QDs complex ································· 9

Figure S9. i-t plots and impedance spectra of various $ZnSO_4$-C_3N_4QDs electrolytes···············10

Figure S10. The binding energy of C_3N_4QD and H_2O upon Zn metal ·······························12

Figure S11. The potential energy change of solvated Zn^{2+} ions passing through the Zn (002) surface at a different distance··13

Figure S12. Typical configuration of the in situ electrochemical AFM cell·························13

Figure S13. XPS analysis of Zn@$ZnSO_4$ and Zn@$ZnSO_4$-C_3N_4QDs································14

Figure S14. The GIXD pattern of pristine Zn···15

Figure S15. SEM morphology of Zn@$ZnSO_4$-C_3N_4QDs after 1st striping process ·················15

Figure S16. SEM morphology of Zn@$ZnSO_4$ after 1st striping process ·····························16

Figure S17. SEM morphology of Zn@$ZnSO_4$-C_3N_4QDs and Zn@$ZnSO_4$ after 1st striping/plating process··16

Figure S18. SEM morphology of Zn@$ZnSO_4$-C_3N_4QDs after 75th striping/plating process ·······17

Figure S19. SEM morphology of Zn@$ZnSO_4$ after 75th striping/plating process ···················17

Figure S20. FIB-SEM images of Zn@$ZnSO_4$ and Zn@$ZnSO_4$-C_3N_4·································18

Figure S21. SEM morphology of Zn@$ZnSO_4$-C_3N_4QDs in different C_3N_4QDs concentrations ···19

Figure S22. Voltage profiles in Zn||SS cells under 2 M $ZnSO_4$ electrolytes ························20

Figure S23. The ion conductivity of various $ZnSO_4$-C_3N_4QDs electrolytes ·························21

Figure S24. Relationship between C_3N_4QDs concentration and double-layer capacitance ·········22

Figure S25. CV curves for Zn||Zn symmetric cells under various $ZnSO_4$-C_3N_4QDs electrolytes··23

Figure S26. CV curves for Zn||Zn symmetric cells under various $ZnSO_4$-C_3N_4QDs electrolytes··24

Figure S27. EIS plots for Zn||Zn symmetric cells in different electrolytes ···························25

Figure S28. EIS plots variation under open-circuit conditions as a function of standing time ·····25

Figure S29. Galvanostatic Zn stipping/plating under various $ZnSO_4$-C_3N_4QDs electrolytes·······26

Figure S30. The thickness variation of Zn||Zn symmetric cells ··27

Figure S31. Galvanostatic Zn stipping/plating under 3 mA cm^{-2} and 5 mA cm^{-2} with 1 mAh cm^{-2}27

Figure S32. Cyclic performance of Zn||V_2O_5 and Zn|C_3N_4QDs| V_2O_5 full cells······················28

Figure S33. Structural and morphology characterization of as-synthesized MnO_2··················28

Figure S34. CV curves of Zn||MnO_2 and Zn|C_3N_4QDs|MnO_2 full cells ······························29

Figure S35. EIS plots of Zn||MnO_2 and Zn|C_3N_4QDs|MnO_2 full cells ································29

Figure S36. Rate performances of Zn||MnO_2 and Zn|C_3N_4QDs|MnO_2 full cells ····················30

Figure S37. Structural and morphology characterization of as-synthesized $VOPO_4$ $2H_2O$ ·········30

Figure S38. EIS plots of Zn||$VOPO_4$ and Zn|C_3N_4QDs|$VOPO_4$ full cells ····························31

Figure S39. Post mortem analysis of the Zn||$VOPO_4$ full cells···31

Figure S40. XPS analysis of $VOPO_4$-cathode after the 15th cycle ······································32

Figure S41. The *ex situ* WAXS patterns in Zn|C_3N_4QDs|$VOPO_4$ full cells ··························32

Figure S42. Open circuit-voltage decay for Zn||$VOPO_4$ and Zn|C_3N_4QDs|$VOPO_4$ full cells·······33

Supplementary Tables

Table S1. Calculated t_{Zn2+} in different $ZnSO_4$-based electrolytes ······································11

Table S2. Theoretical percentage of the Zn^{2+}ions that interacted with C_3N_4QDs in 2M $ZnSO_4$ ···14

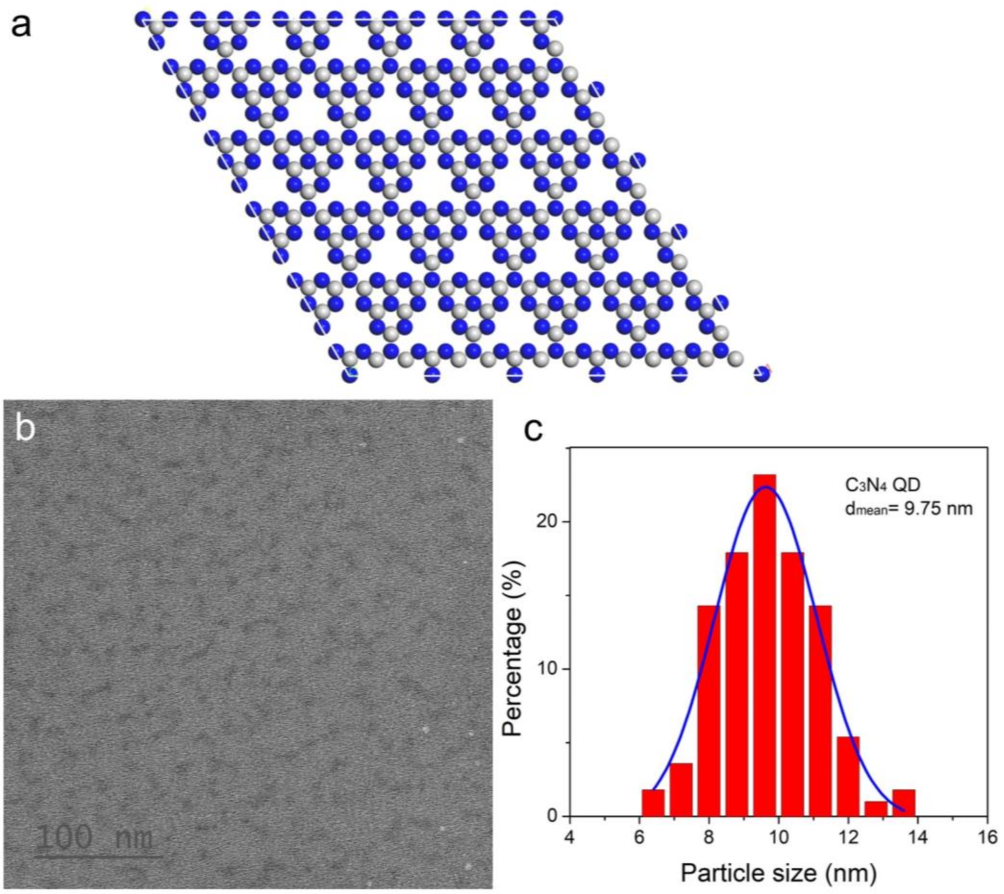

Supplementary Figure 1. (a) Typical structure of the C_3N_4QDs, the grey, and blue balls represent C and N atoms, respectively. (b) TEM images of the as-synthesized C_3N_4QDs, and (c) the corresponding size distribution of C_3N_4QDs.

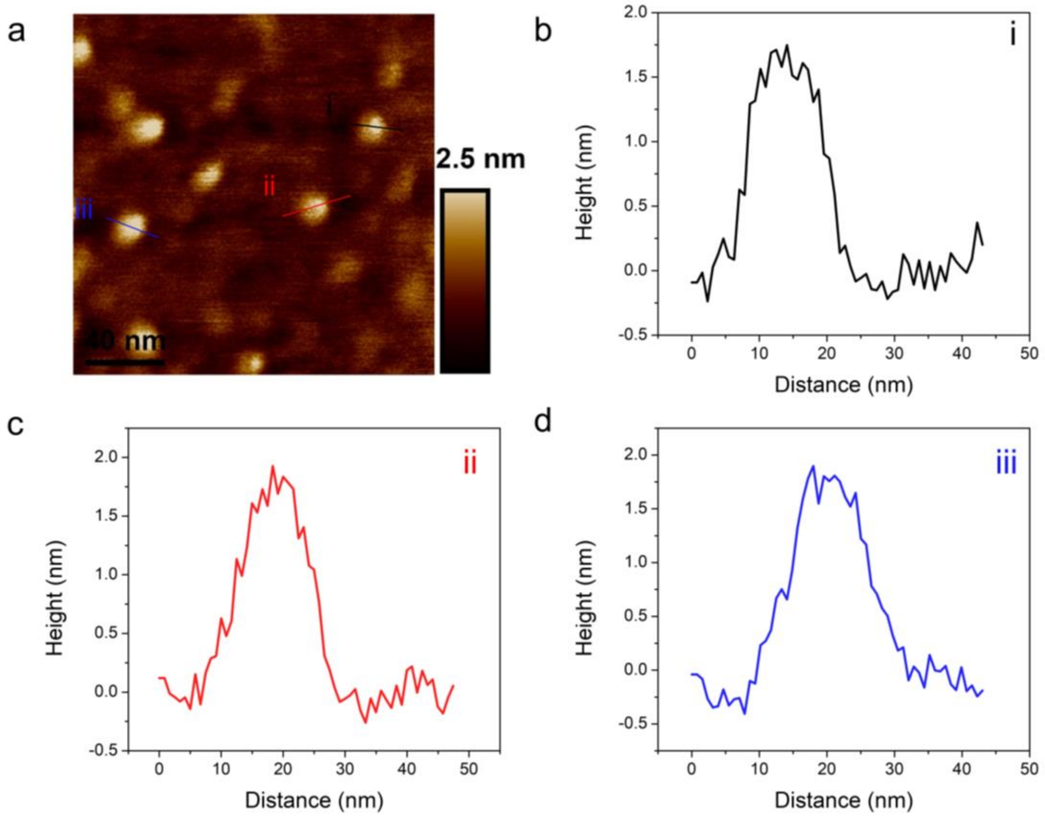

Supplementary Figure 2. (a) AFM height images of the as-synthesized C_3N_4QDs, (b-d) the height profile of the corresponding line.

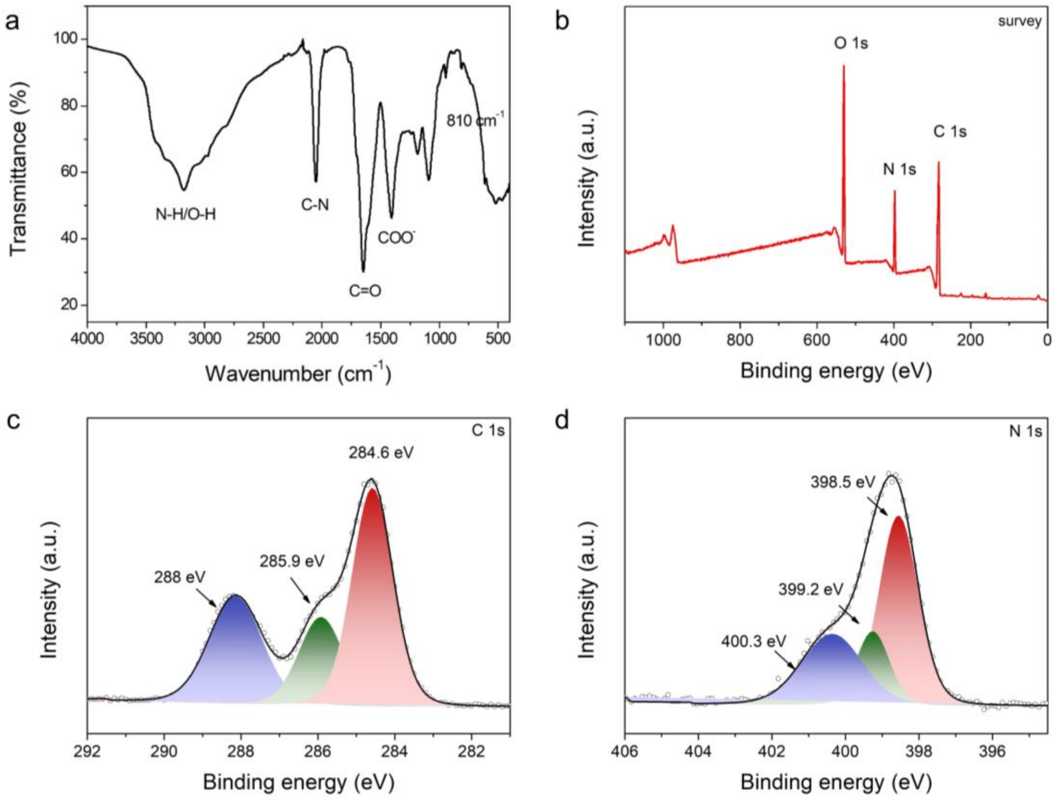

Supplementary Figure 3. (a) FT-IR spectroscopy of the as-synthesized C_3N_4QDs, (b) XPS survey spectra and high-resolution (c) C 1s, (d) N 1s, spectra of the as-synthesized C_3N_4QDs.

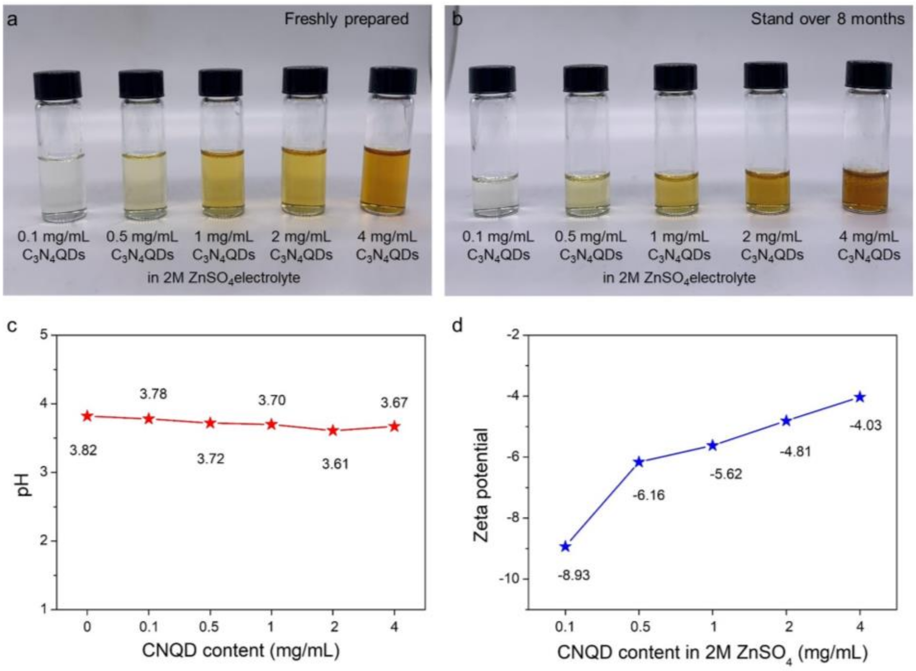

Supplementary Figure 4. (a) Photographs of the fresh prepared $ZnSO_4$ aqueous electrolytes with different C_3N_4QDs contents, (b) Photographs of the $ZnSO_4$-C_3N_4QDs aqueous electrolytes after standing over 8 months, (c) pH value of $ZnSO_4$-C_3N_4QDs aqueous electrolytes with varying concentration of C_3N_4QDs, (d) Zeta potential of $ZnSO_4$-C_3N_4QDs aqueous electrolytes with varying concentration of C_3N_4QDs.

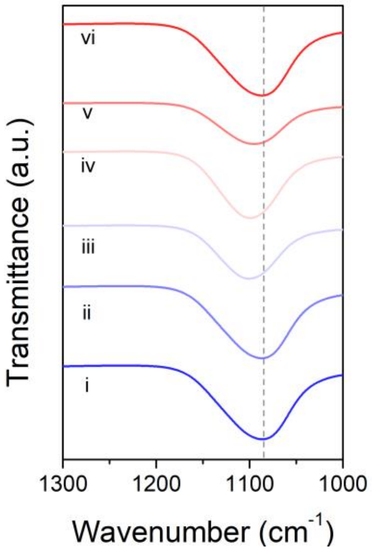

Supplementary Figure 5. Typical FT-IR spectra of (i) 2M $ZnSO_4$, (ii) 2M $ZnSO_4$ + 0.1 mg ml^{-1} C_3N_4QDs, (iii) 2M $ZnSO_4$ + 0.5 mg ml^{-1} C_3N_4QDs, (iv) 2M $ZnSO_4$ + 1 mg ml^{-1} C_3N_4QDs, (v) 2M $ZnSO_4$ + 2 mg ml^{-1} C_3N_4QDs, (vi) 2M $ZnSO_4$ + 4 mg ml^{-1} C_3N_4QDs in the region of 1000 - 1300 cm^{-1}.

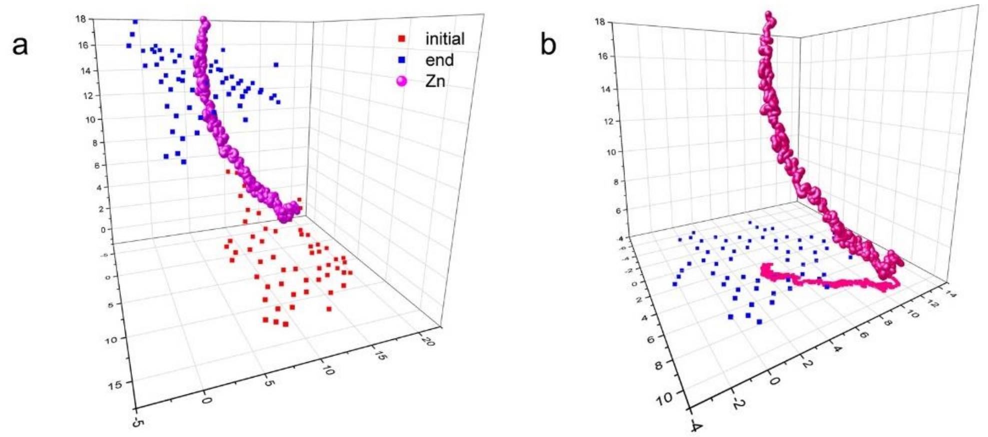

Supplementary Figure 6. (a) The position motion track and (b) projection motion track of Zn^{2+} ions relative to C_3N_4QD after 30000 fs in MD simulation.

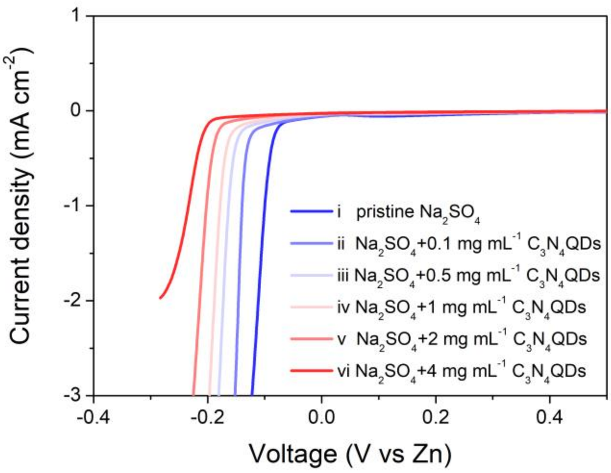

Supplementary Figure 7. Hydrogen evolution reaction (HER) in H_2O with 2 M Na_2SO_4 as supporting salt measured in a Zn || Stainsteel cell using linear potential scan at a scan rate of 1 mV s^{-1}.

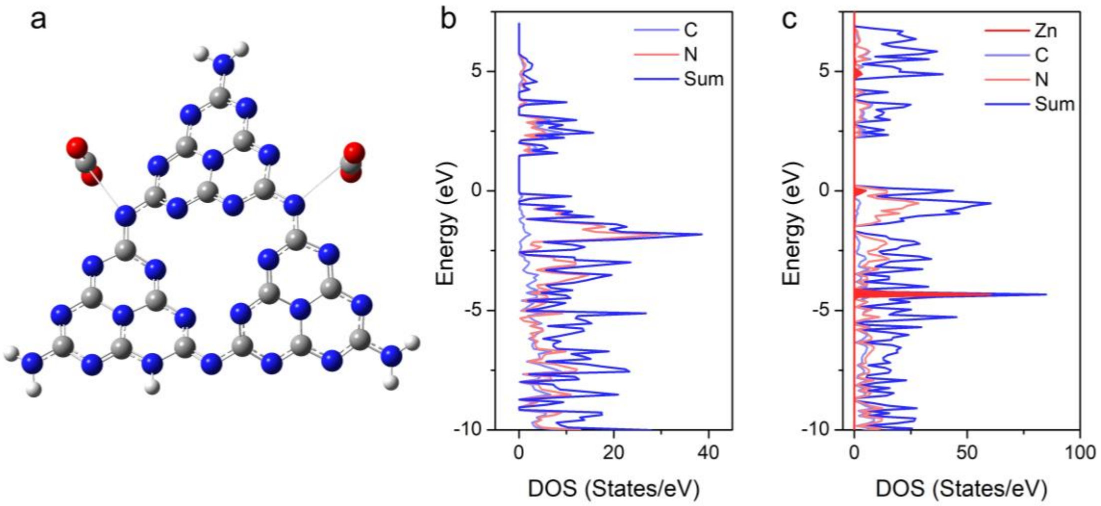

Supplementary Figure 8. (a) The periodic unit of the C_3N_4QDs, the grey, blue, red, and white balls represent C, N, O, H, and S atoms, respectively. Calculated total DOS of (b) C_3N_4QDs and (c) Zn adsorbed C_3N_4QDs complex.

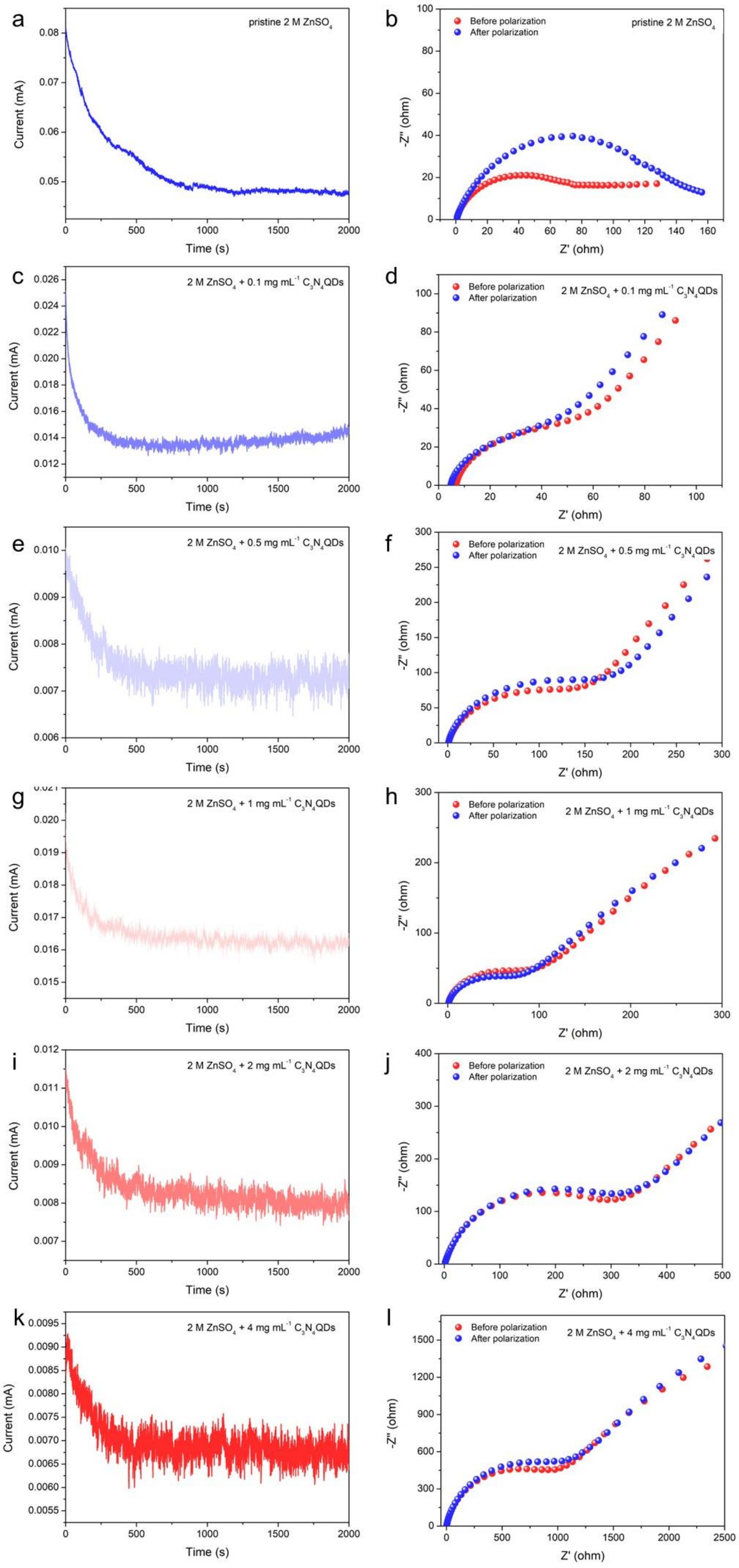

Supplementary Figure 9. (a) Current-time plots of Zn symmetric cell in various electrolytes after polarization at a constant potential (25 mV) for 2000 s, (b) the impedance spectra before and after the polarization.

The transference number of Zn^{2+} (t_{Zn2+}) was determined by the following equation:

$$t_{Zn^{2+}} = \frac{I_s(\Delta V - I_0 R_0)}{I_0(\Delta V - I_s R_s)}$$

where ΔV is the applied voltage (25 mV), I_0 and R_0 are the initial current and resistance, respectively; I_s and R_s are the steady-state current and resistance, respectively.

Supplementary Table 1. Calculated t_{Zn2+} in different $ZnSO_4$-based electrolytes.

electrolytes	t_{Zn2+}
2M $ZnSO_4$	0.577
2M $ZnSO_4$ + 0.1 mg ml^{-1} C_3N_4QDs	0.610
2M $ZnSO_4$ + 0.5 mg ml^{-1} C_3N_4QDs	0.712
2M $ZnSO_4$ + 1 mg ml^{-1} C_3N_4QDs	0.796
2M $ZnSO_4$ + 2 mg ml^{-1} C_3N_4QDs	0.674
2M $ZnSO_4$ + 4 mg ml^{-1} C_3N_4QDs	0.682

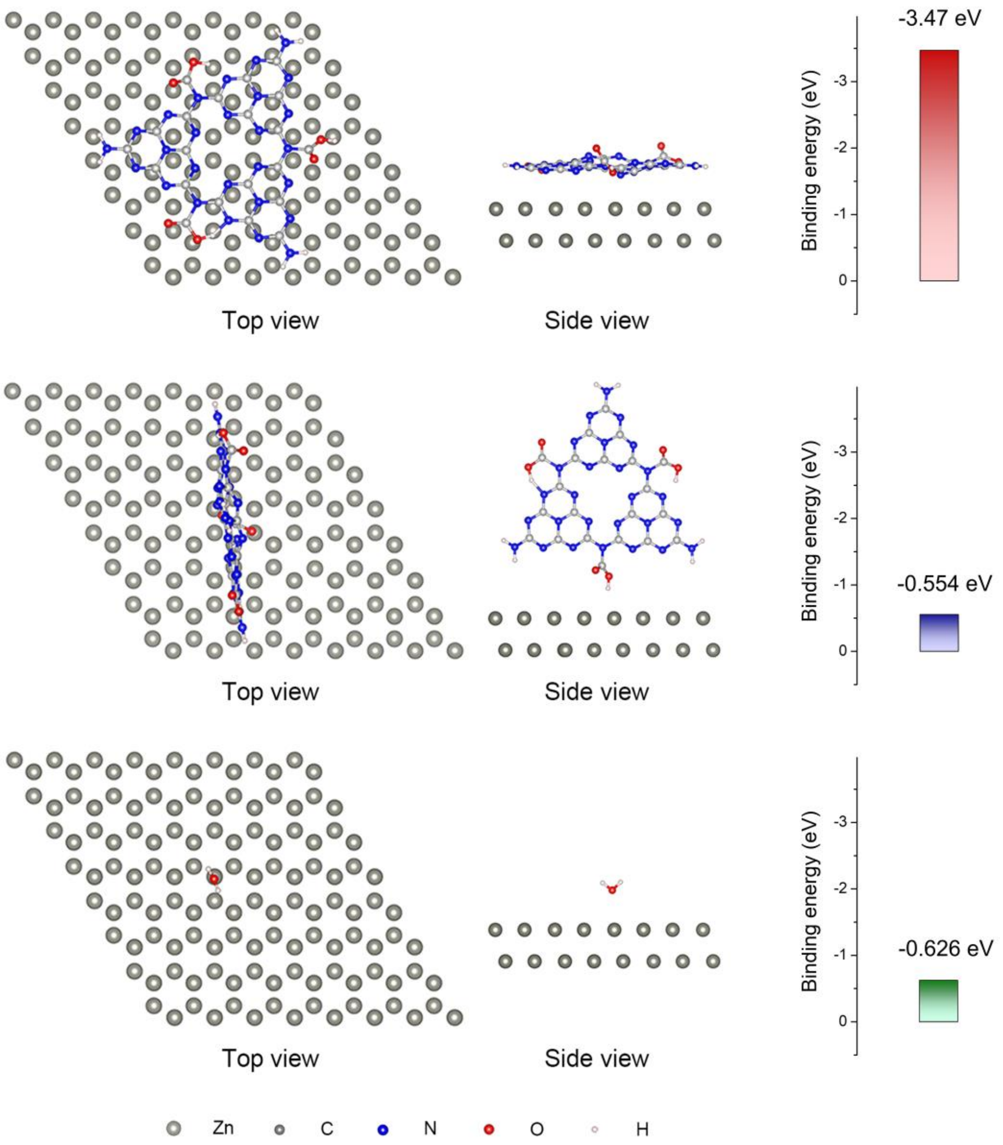

Supplementary Figure 10. Geometric structures and binding energy of (002) facets for Zn metal with C_3N_4QDs or H_2O molecules; The big grey, small light grey, blue, red, and pink balls represent Zn, C, N, O, and H atoms, respectively

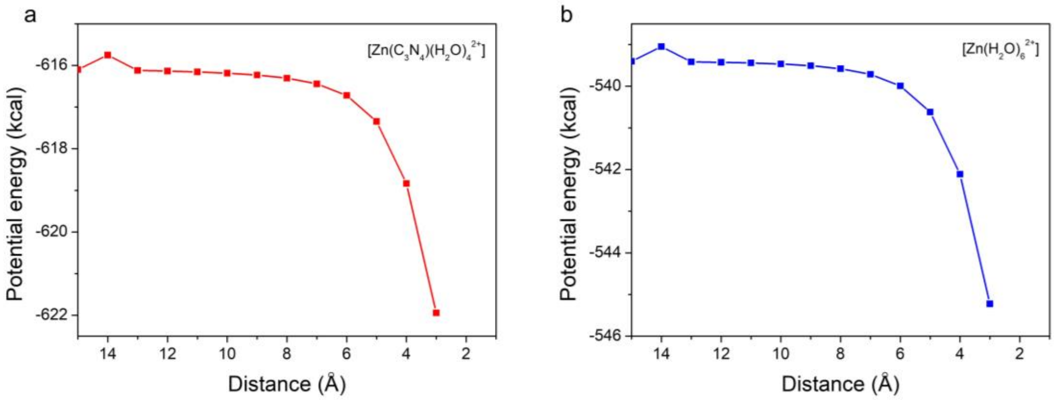

Supplementary Figure 11. The potential energy change of the solvated Zn^{2+} ions passing through the Zn (002) surface at a different distance.

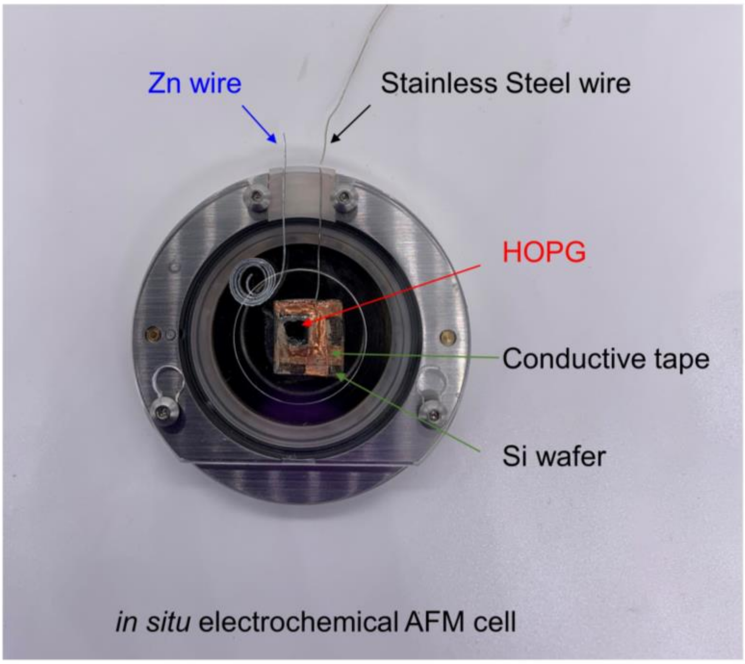

Supplementary Figure 12. Typical configuration of the in situ electrochemical AFM cell.

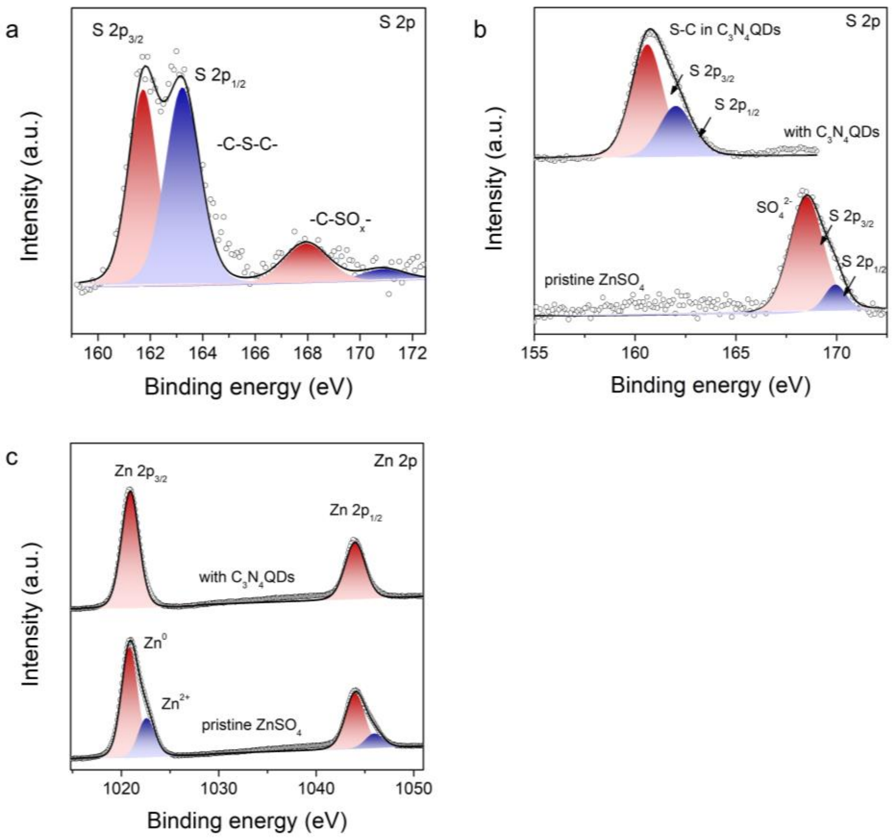

Supplementary Figure 13. High-resolution XPS analyzation of the S2p peaks of (a) C_3N_4QDs, and (b) Zn anode after 15th stripping/plating process in 2 M $ZnSO_4$ + 0.5 mg mL^{-1} C_3N_4QDs electrolyte (top spectrum) and conventional 2 M $ZnSO_4$ electrolyte (bottom spectrum); (c) High-resolution XPS analyzation of the Zn2p peaks obtained from Zn anode after 15th stripping/plating process in 2 M $ZnSO_4$ + 0.5 mg mL^{-1} C_3N_4QDs electrolyte (top spectrum) and conventional 2 M $ZnSO_4$ electrolyte (bottom spectrum).

Supplementary Table 2. The percentage of the Zn^{2+}ions that interacted with C_3N_4QDs in 2M $ZnSO_4$ theoretically.

The concentration of C_3N_4QDs	The percentage of the Zn^{2+}ions that interacted with C_3N_4QDs
0.1 mg mL^{-1}	0.013%
0.5 mg mL^{-1}	0.063%
1 mg mL^{-1}	0.125%
2 mg mL^{-1}	0.251%
3 mg mL^{-1}	0.376%

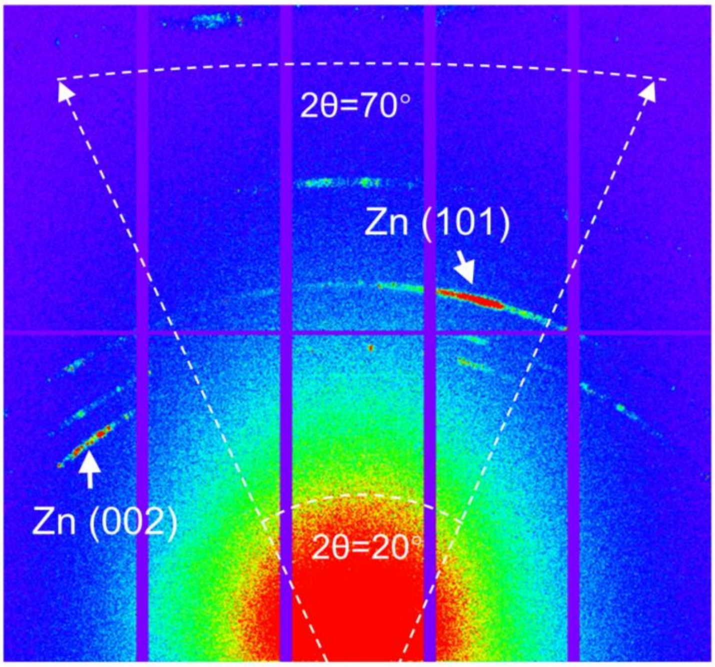

Supplementary Figure 14. 2D synchrotron grazing-incidence X-ray diffraction (GIXD) pattern of pristine Zn.

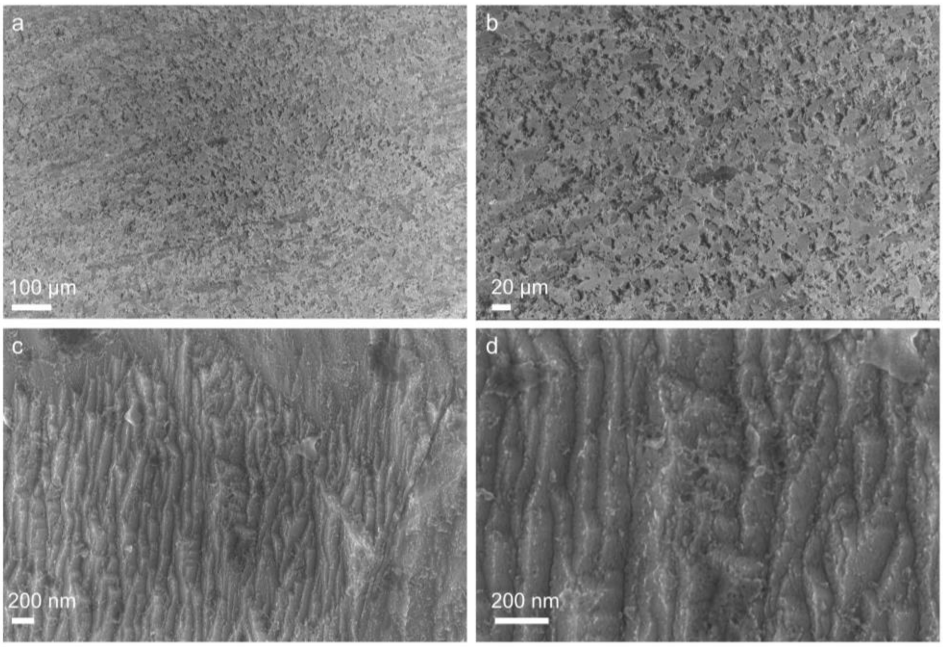

Supplementary Figure 15. SEM morphology of Zn@$ZnSO_4$-C_3N_4QDs after 1st striping process.

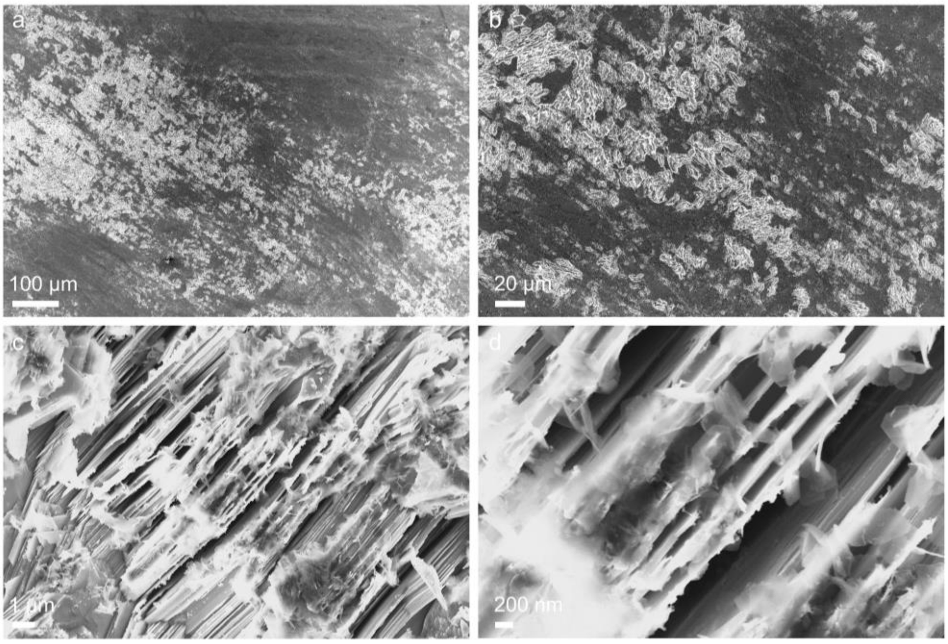

Supplementary Figure 16. SEM morphology of Zn@$ZnSO_4$ after 1st striping process.

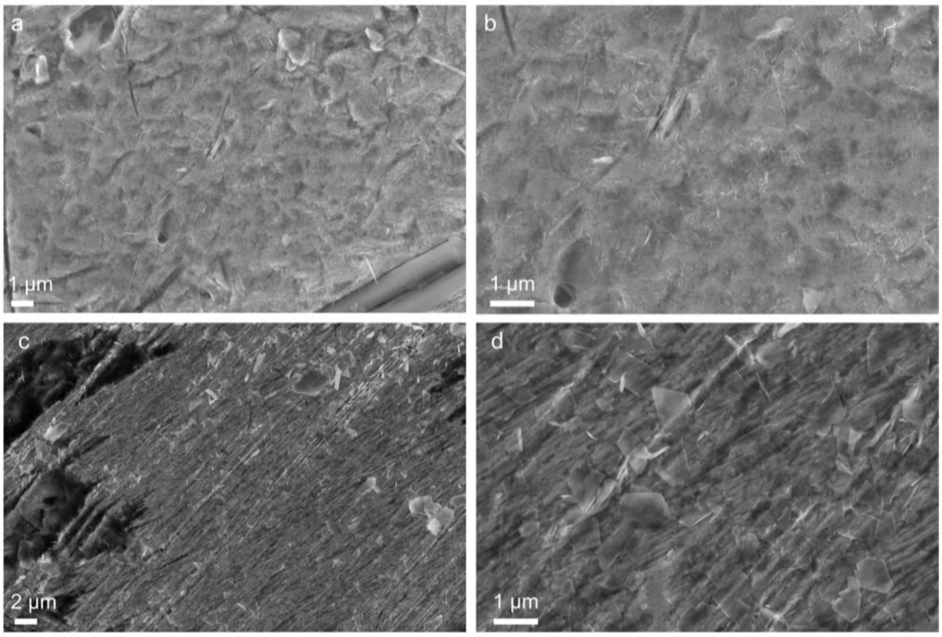

Supplementary Figure 17. SEM morphology of (a, b) Zn@$ZnSO_4$-C_3N_4QDs and (c, d) Zn@$ZnSO_4$ after 1st striping/plating process.

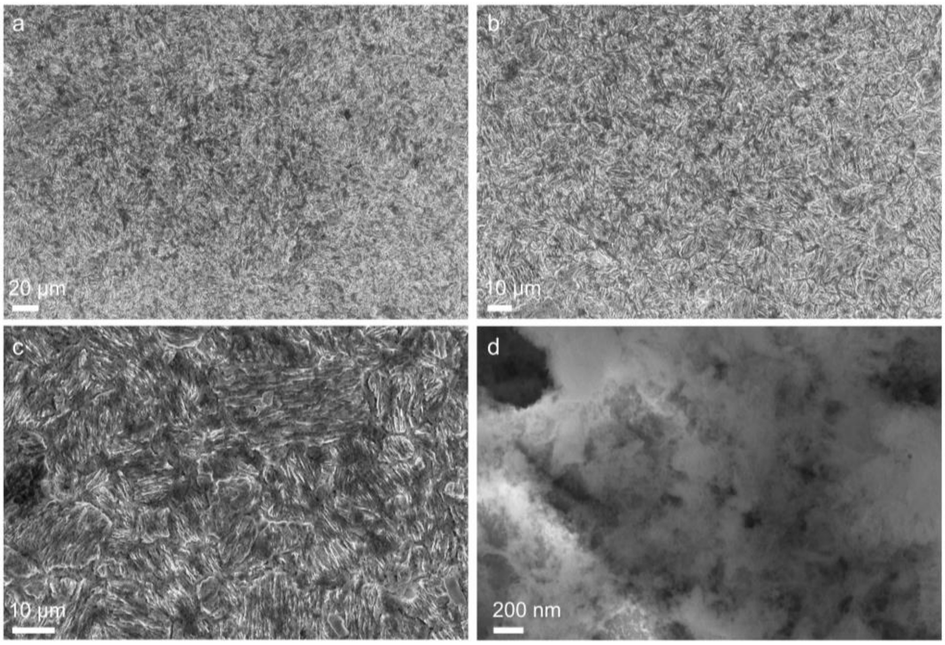

Supplementary Figure 18. SEM morphology of $Zn@ZnSO_4$-C_3N_4QDs after 75th striping/plating process.

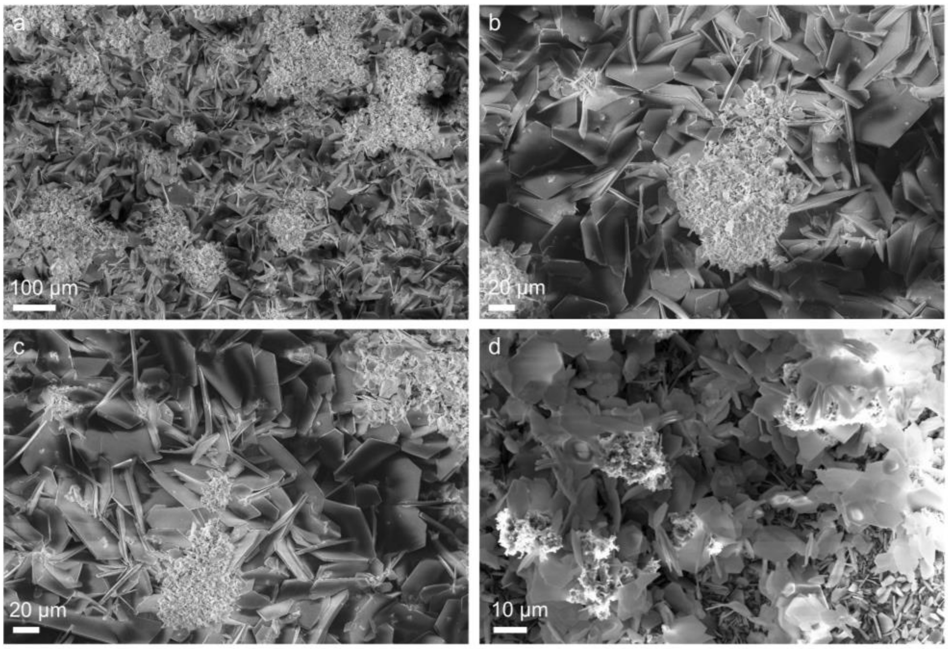

Supplementary Figure 19. SEM morphology of $Zn@ZnSO_4$ after 75th striping/plating process.

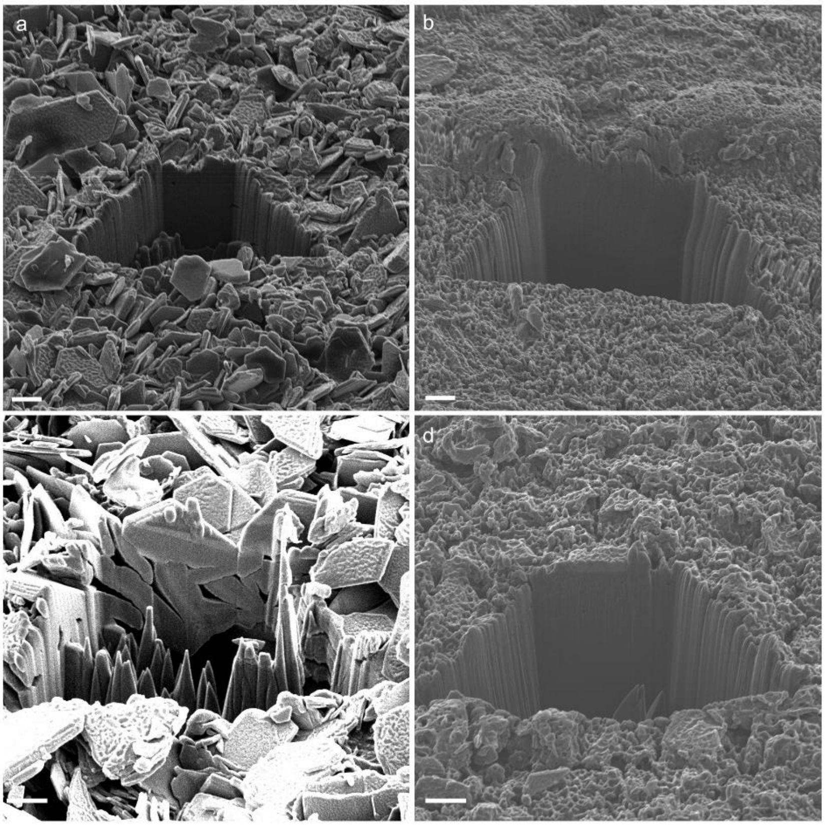

Supplementary Figure 20. Focused ion beam (FIB)-SEM images of (a) Zn@$ZnSO_4$ after 15 cycles, (b) Zn@$ZnSO_4$-C_3N_4 after 15 cycles, (c) Zn@$ZnSO_4$ after 50 cycles, (d) Zn@$ZnSO_4$-C_3N_4 after 50 cycles, respectively. Scale bar: 2 μm for (a), (b), (c), (d).

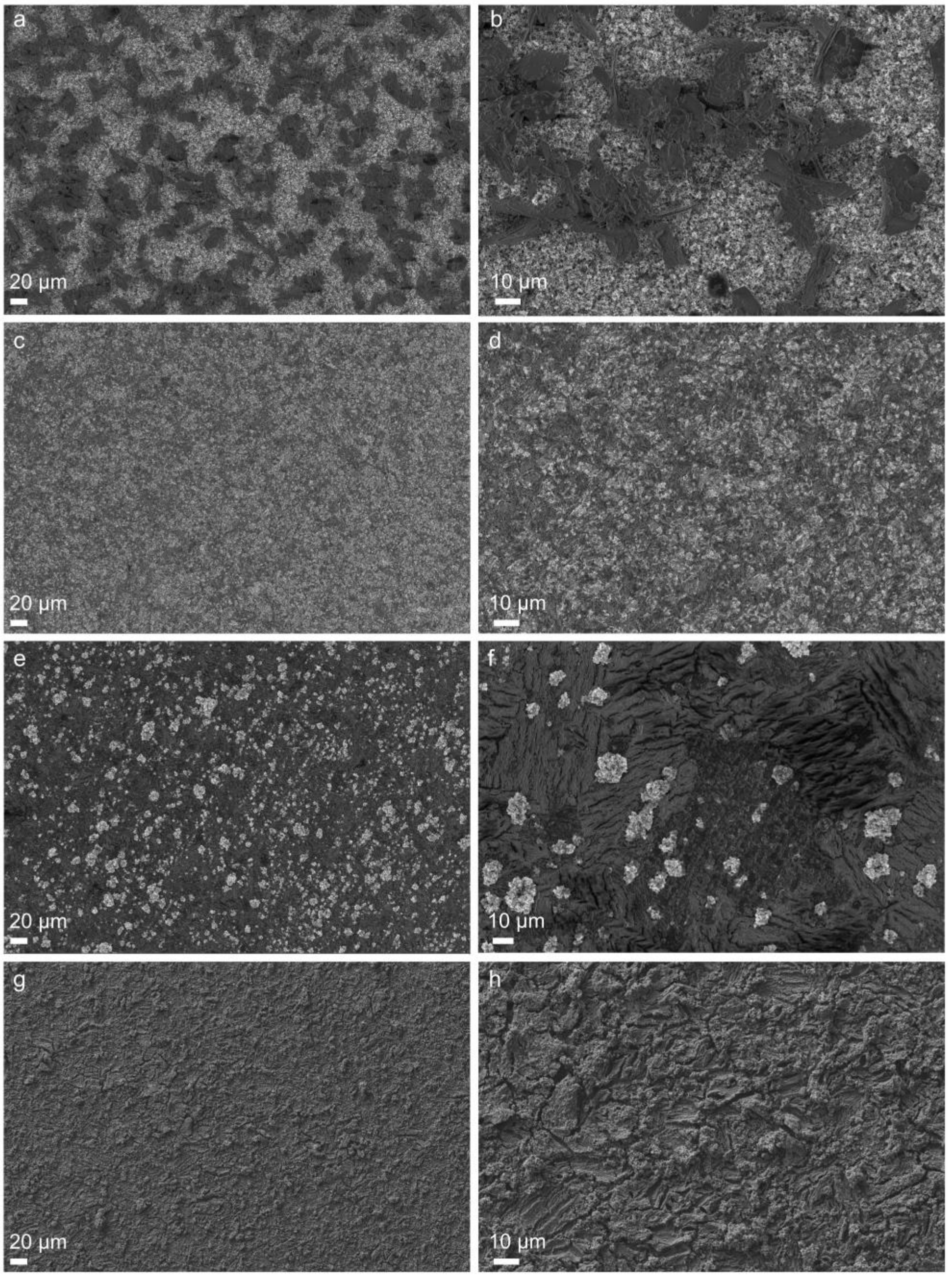

Supplementary Figure 21. SEM morphology of Zn@$ZnSO_4$-C_3N_4QDs in different C_3N_4QDs concentrations after 75th striping/plating process, (a, b) 2M $ZnSO_4$ + 0.1 mg ml^{-1} C_3N_4QDs, (c, d) 2M $ZnSO_4$ + 1 mg ml^{-1} C_3N_4QDs, (e, f) 2M $ZnSO_4$ + 2 mg ml^{-1} C_3N_4QDs, (g, h) 2M $ZnSO_4$ + 4 mg ml^{-1} C_3N_4QDs.

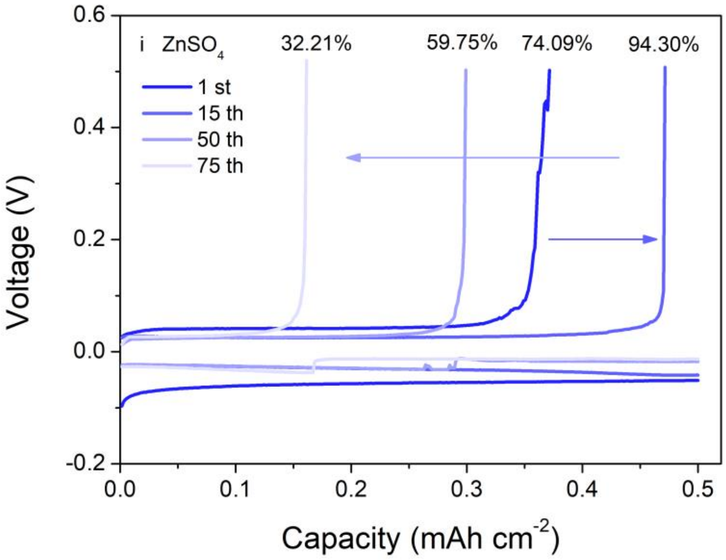

Supplementary Figure 22. Voltage profiles obtained in 2 M $ZnSO_4$ electrolytes at various cycles in Zn||SS cells.

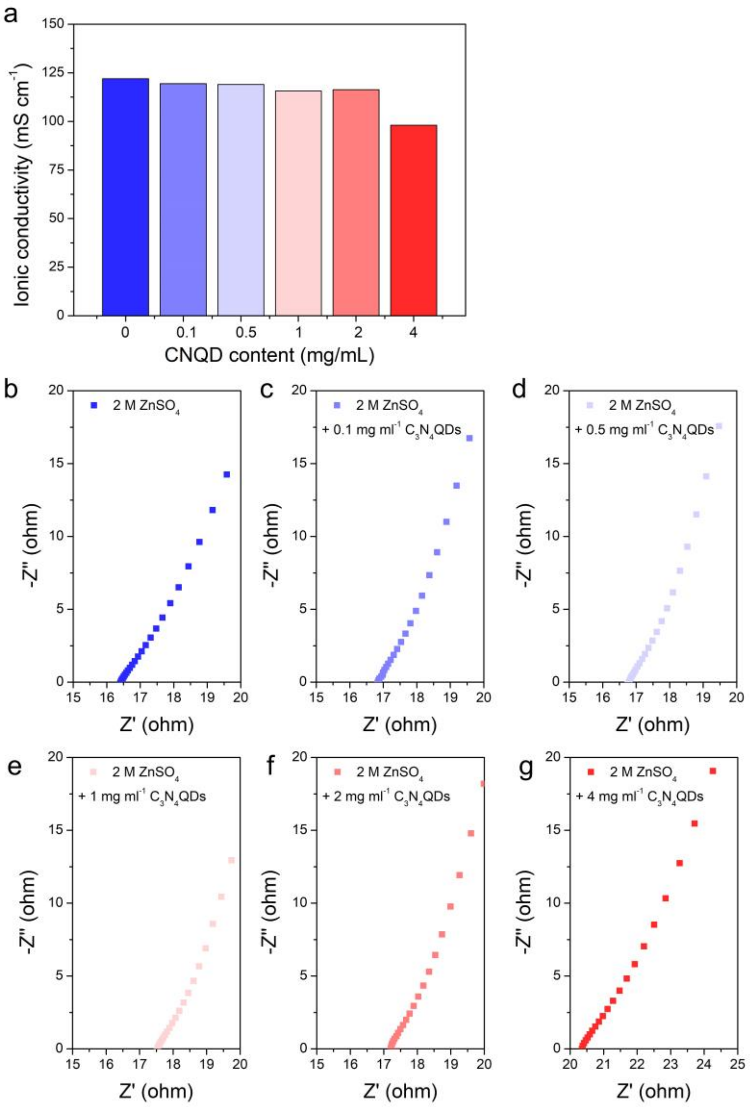

Supplementary Figure 23. (a) Relationship between C_3N_4QDs concentration and ion conductivity of the $ZnSO_4$-C_3N_4QDs electrolyte systems. Nyquist plots collected at open circuit voltage (OCV) over the frequency range of 100 kHz to 1 Hz in a beaker cell with two Pt foil electrode under (b) 2M $ZnSO_4$, (c) 2M $ZnSO_4$ + 0.1 mg ml^{-1} C_3N_4QDs, (d) 2M $ZnSO_4$ + 0.5 mg ml^{-1} C_3N_4QDs, (e) 2M $ZnSO_4$ + 1 mg ml^{-1} C_3N_4QDs, (f) 2M $ZnSO_4$ + 2 mg ml^{-1} C_3N_4QDs, (g) 2M $ZnSO_4$ + 4 mg ml^{-1} C_3N_4QDs electrolytes.

A beaker cell is employed, and Pt foil (1 cm^2 in area) is used as the working electrode and another Pt foil (1 cm^2 in area) as the reference and counter electrode. The ionic conductivity of the electrolyte can be calculated via the following equation:

$$\sigma = \frac{L}{R_b S}$$

in which L is the distance between the two electrodes (2 cm), S is the contact area (1 cm^2), and R_b is the bulk resistance.

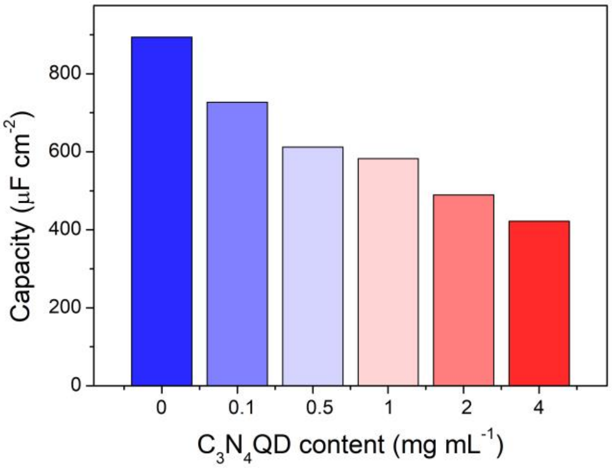

Supplementary Figure 24. Relationship between C_3N_4QDs concentration and double-layer capacitance of the $ZnSO_4$-C_3N_4QDs electrolyte systems.

The double-layer capacitance is calculated from the following equations,

$$C = i_c/v$$

The linear dependence of the capacitive current (i_c) on scan rate (v) can be used to determine the capacity. Capacity (C) is obtained from the slope of the i_c versus v profiles. Here, we choose $i_c = (i_{0v+} - i_{0v-})/2$. It is the half value of the current difference during the forward scan and negative scan at 0 V

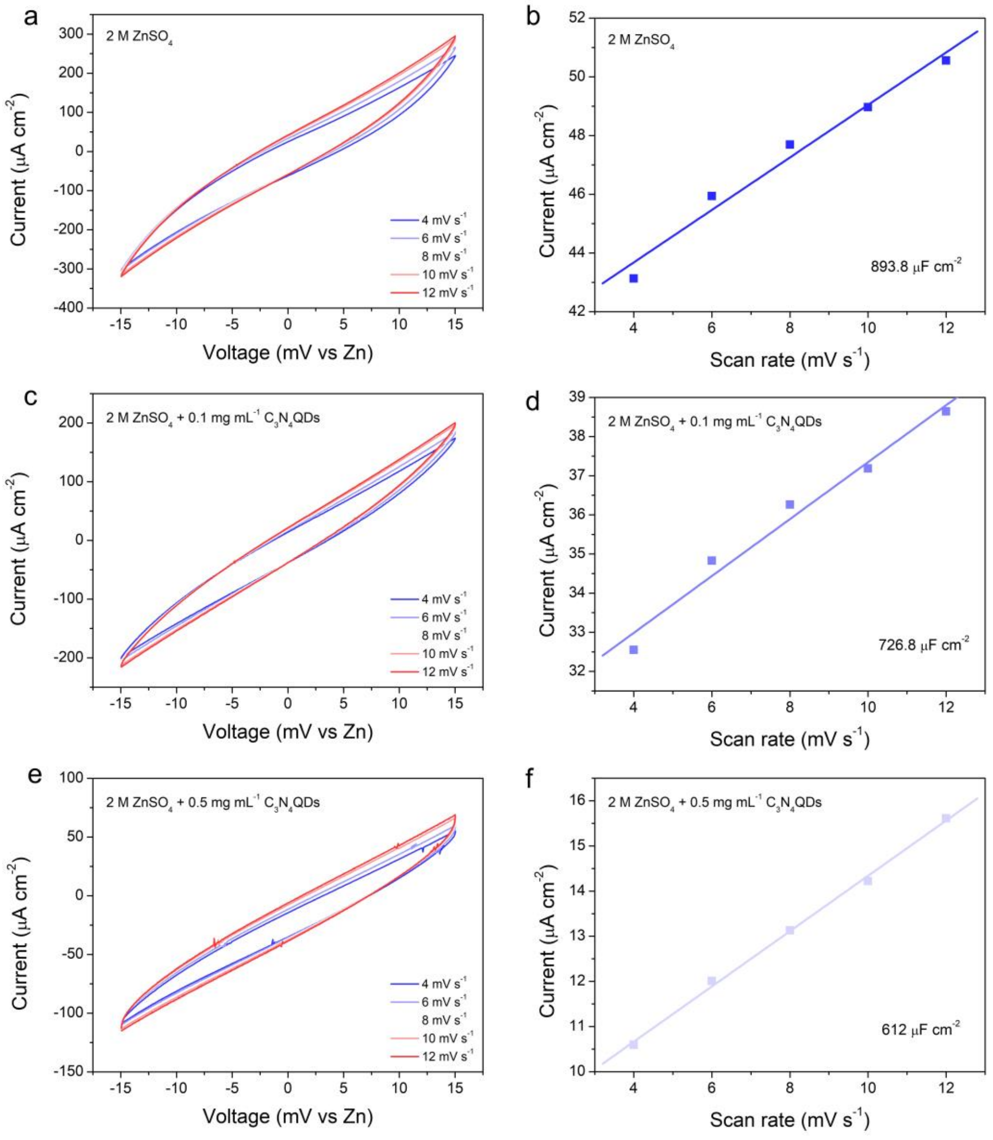

Supplementary Figure 25. Double layer capacity measurements for Zn substrates in $ZnSO_4$-C_3N_4QDs electrolyte systems. (a) Cyclic voltammograms curves for Zn || Zn symmetric coin cells in a voltage range of −15 mV to 15 mV under various scanning rates in 2M $ZnSO_4$ electrolyte. (b) Plots of capacitive currents versus scan rates in 2M $ZnSO_4$ electrolyte. (c) Cyclic voltammograms curves for Zn || Zn symmetric coin cells in a voltage range of –15 mV to 15 mV under various scanning rates in 2M $ZnSO_4$ + 0.1 mg ml^{-1} C_3N_4QDs electrolyte. (d) Plots of capacitive currents versus scan rates in 2M $ZnSO_4$ + 0.1 mg ml^{-1} C_3N_4QDs electrolyte. (e) Cyclic voltammograms curves for Zn || Zn symmetric coin cells in a voltage range of –15 mV to 15 mV under various scanning rates in 2M $ZnSO_4$ + 0.5 mg ml^{-1} C_3N_4QDs electrolyte. (f) Plots of capacitive currents versus scan rates in 2M $ZnSO_4$ + 0.5 mg ml^{-1} C_3N_4QDs electrolyte.

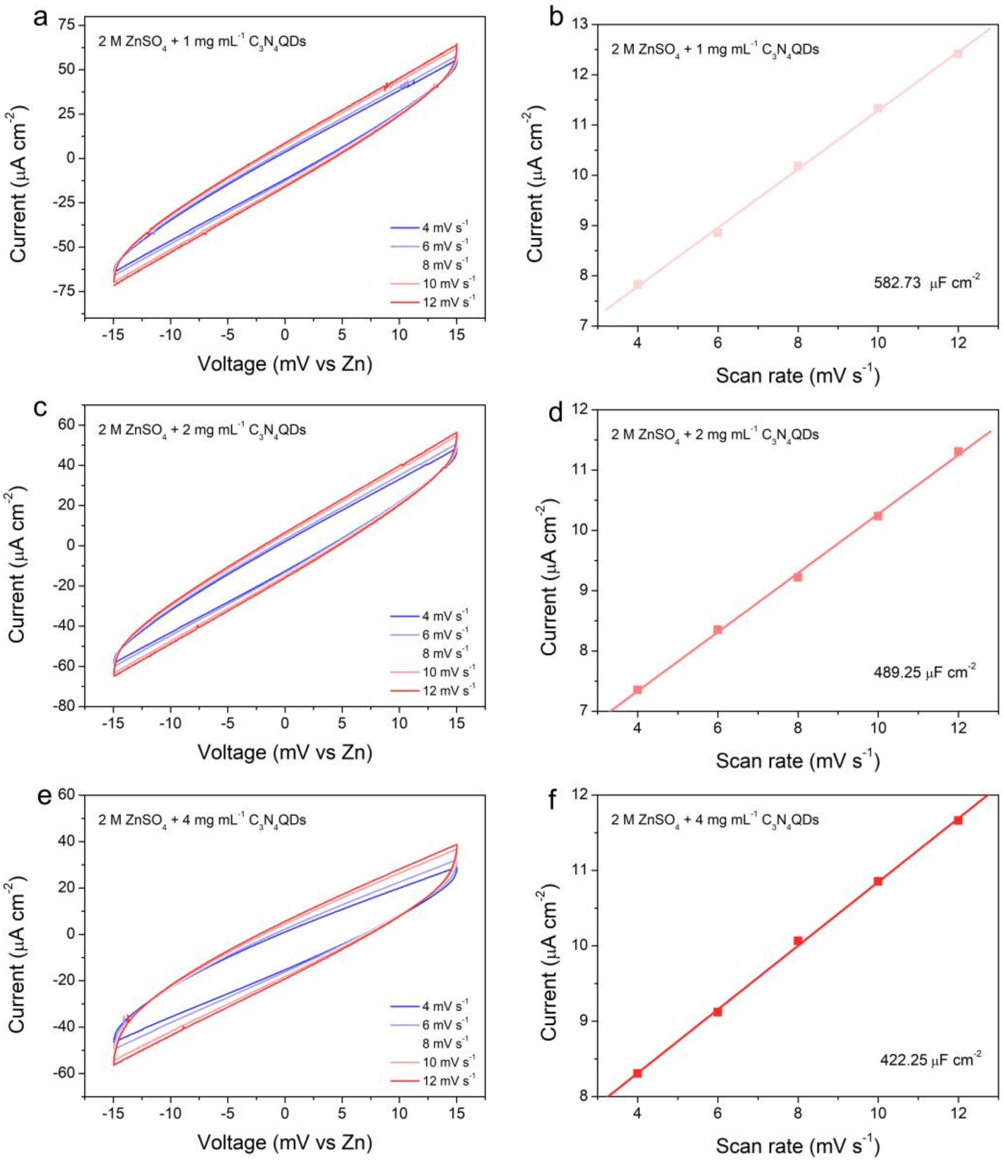

Supplementary Figure 26. Double layer capacity measurements for Zn substrates in $ZnSO_4$-C_3N_4QDs electrolyte systems. (a) Cyclic voltammograms curves for Zn || Zn symmetric coin cells in a voltage range of –15 mV to 15 mV under various scanning rates in 2M $ZnSO_4$ +1 mg ml^{-1} C_3N_4QDs electrolyte. (b) Plots of capacitive currents versus scan rates in 2M $ZnSO_4$ + 1 mg ml^{-1} C_3N_4QDs electrolyte. (c) Cyclic voltammograms curves for Zn || Zn symmetric coin cells in a voltage range of –15 mV to 15 mV under various scanning rates in 2M $ZnSO_4$ + 2 mg ml^{-1} C_3N_4QDs electrolyte. (d) Plots of capacitive currents versus scan rates in 2M $ZnSO_4$ + 2 mg ml^{-1} C_3N_4QDs electrolyte. (e) Cyclic voltammograms curves for Zn || Zn symmetric coin cells in a voltage range of –15 mV to 15 mV under various scanning rates in 2M $ZnSO_4$ + 4 mg ml^{-1} C_3N_4QDs electrolyte. (f) Plots of capacitive currents versus scan rates in 2M $ZnSO_4$ + 4 mg ml^{-1} C_3N_4QDs electrolyte.

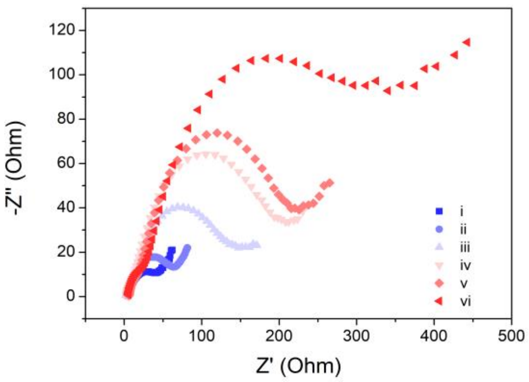

Supplementary Figure 27. Comparison of EIS plots for Zn||Zn symmetric cells in different electrolytes. (i) 2 M $ZnSO_4$, (ii) 2 M $ZnSO_4$ + 0.1 mg ml^{-1} C_3N_4QDs, (iii) 2 M $ZnSO_4$ + 0.5 mg ml^{-1} C_3N_4QDs, (iv) 2 M $ZnSO_4$ + 1 mg ml^{-1} C_3N_4QDs, (v) 2 M $ZnSO_4$ + 2 mg ml^{-1} C_3N_4QDs, (vi) 2 M $ZnSO_4$ + 4 mg ml^{-1} C_3N_4QDs.

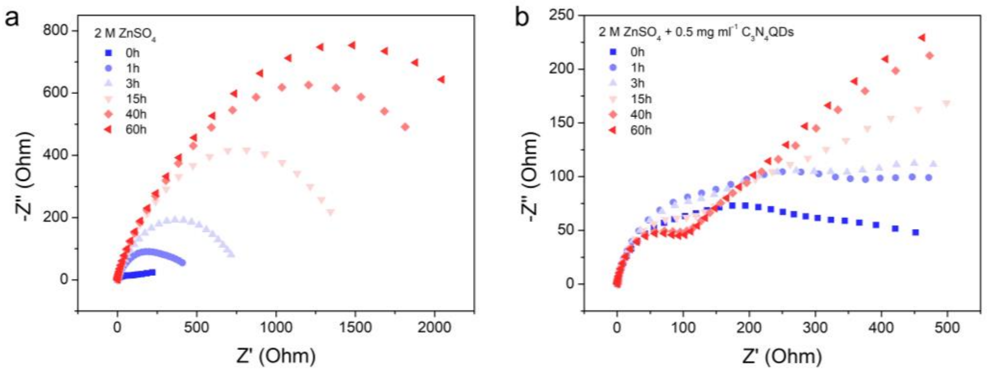

Supplementary Figure 28. EIS plots of Zn||Zn cells in (a) 2M $ZnSO_4$ + 0.5 mg ml^{-1} C_3N_4QDs, and (b) 2M $ZnSO_4$ under open-circuit conditions as a function of standing time.

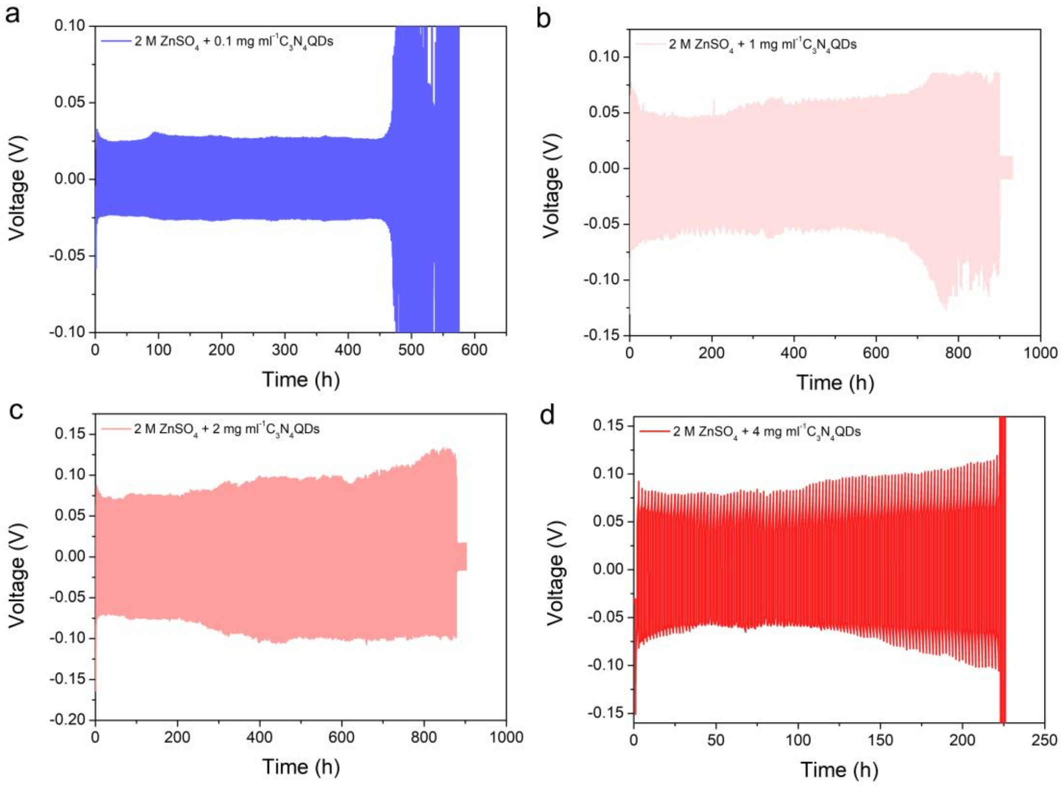

Supplementary Figure 29. Long-term galvanostatic Zn stipping/plating in the Zn||Zn symmetric cells. Time-voltage profiles of (a) 2 M $ZnSO_4$ + 0.1 mg ml^{-1} C_3N_4QDs, (b) 2 M $ZnSO_4$ + 1 mg ml^{-1} C_3N_4QDs, (c) 2 M $ZnSO_4$ + 2 mg ml^{-1} C_3N_4QDs, and (d) 2 M $ZnSO_4$ + 4 mg ml^{-1} C_3N_4QDs under the current density of 1 mA cm^{-2} and the areal capacity of 1 mAh cm^{-2}.

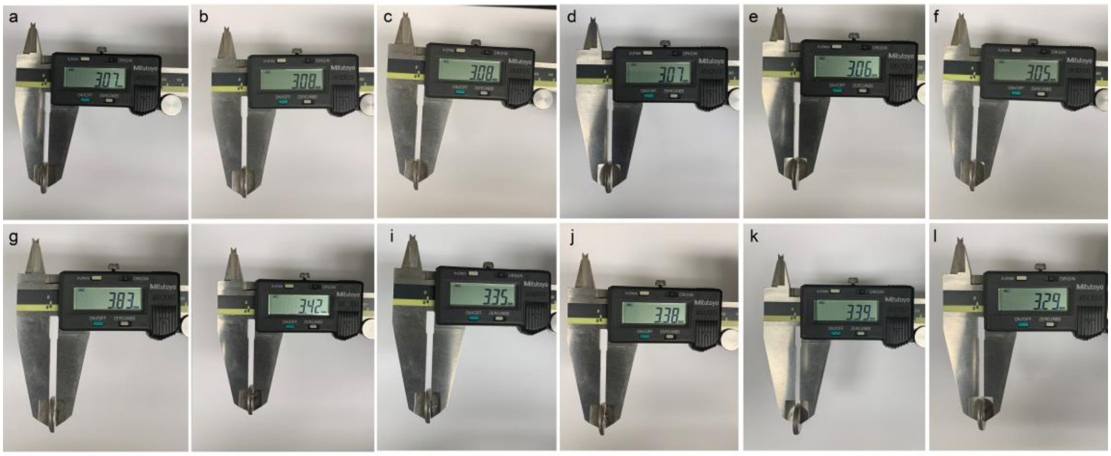

Supplementary Figure 30. The thickness variation of Zn||Zn symmetric cells after Zn stripping/plating. The freshly assembled Zn||Zn symmetric cells with (a) 2 M $ZnSO_4$, (b) 2 M $ZnSO_4$ + 0.1 mg ml^{-1} C_3N_4QDs, (c) 2 M $ZnSO_4$ + 0.5 mg ml^{-1} C_3N_4QDs, (d) 2 M $ZnSO_4$ + 1 mg ml^{-1} C_3N_4QDs, (e) 2 M $ZnSO_4$ + 2 mg ml^{-1} C_3N_4QDs, and (f) 2 M $ZnSO_4$ + 4 mg ml^{-1} C_3N_4QDs; the corresponding thickness of Zn||Zn symmetric cells after cycling under the current density of 1 mA cm^{-2} and the areal capacity of 1 mAh cm^{-2}, (a) 170 h in 2 M $ZnSO_4$, (b) 565 h in 2 M $ZnSO_4$ + 0.1 mg ml^{-1} C_3N_4QDs, (c) 1200 h in 2 M $ZnSO_4$ + 0.5 mg ml^{-1} C_3N_4QDs, (d) 900 h in 2 M $ZnSO_4$ + 1 mg ml^{-1} C_3N_4QDs, (e) 880 h in 2 M $ZnSO_4$ + 2 mg ml^{-1} C_3N_4QDs, and (f) 225 h in 2 M $ZnSO_4$ + 4 mg ml^{-1} C_3N_4QDs.

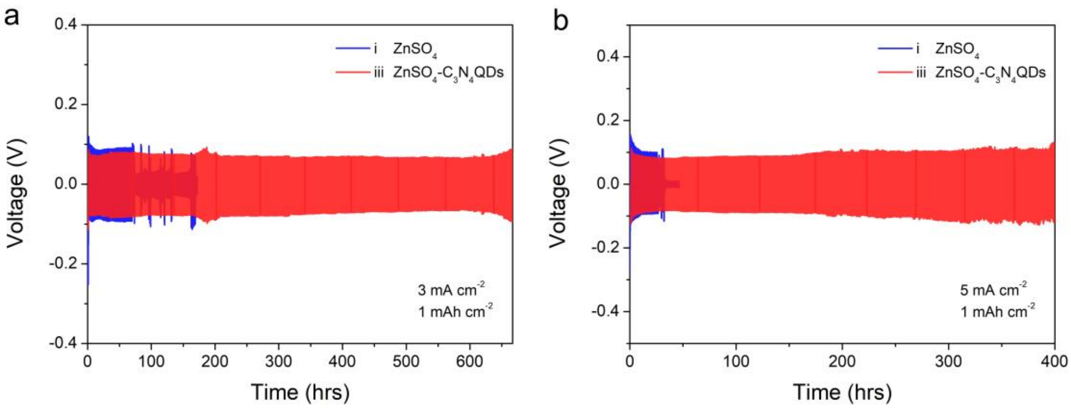

Supplementary Figure 31. Comparison of long-term galvanostatic Zn stripping/plating in the Zn||Zn symmetric cells and time-voltage profiles under (a) 3 mA cm^{-2} and (b) 5 mA cm^{-2} with 1 mAh cm^{-2}.

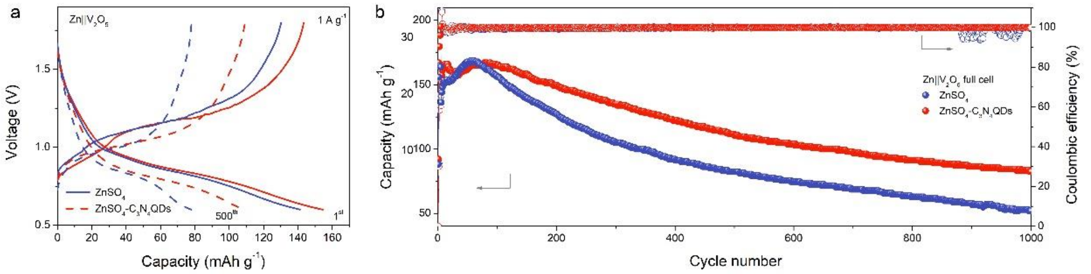

Supplementary Figure 32. (a) Typical voltage profiles of 1st and 500th cycles of Zn||V_2O_5 and Zn|C_3N_4QDs| V_2O_5 batteries at 1 A g^{-1}; (b) Cyclic performance of Zn||V_2O_5 and Zn|C_3N_4QDs| V_2O_5 batteries at 1 A g^{-1}.

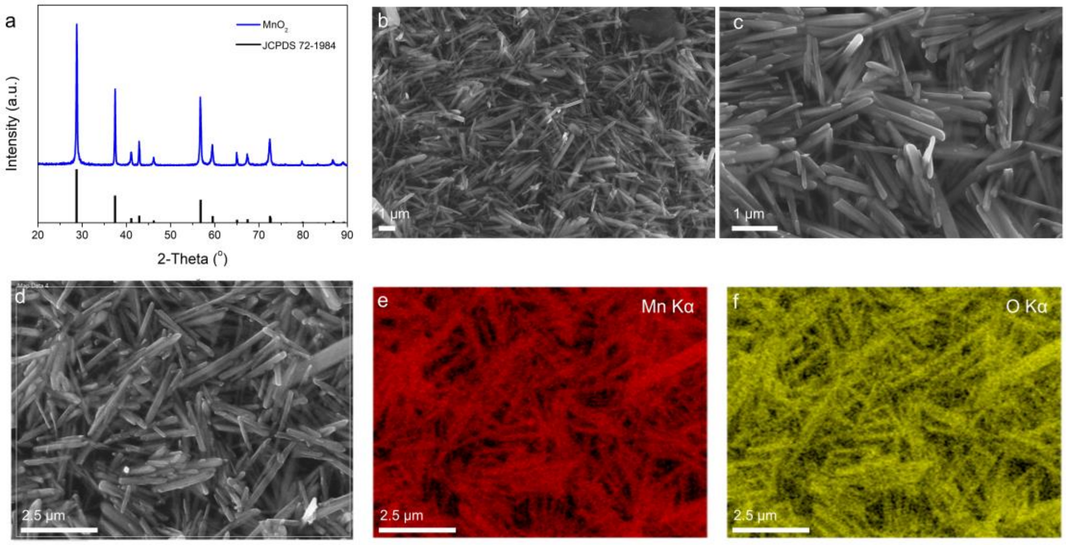

Supplementary Figure 33. Structural and morphology characterization of as-synthesized MnO_2. (a) XRD pattern of the synthesized MnO_2, (b – d) SEM morphology of the synthesized MnO_2, (e – f) Energy dispersive spectroscopy (EDS) mapping of Mn and O components in the synthesized MnO_2.

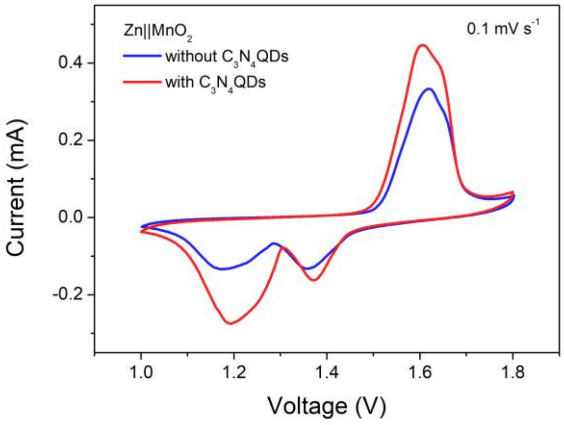

Supplementary Figure 34. Comparison of electrochemical performance of Zn||MnO_2 batteries in 2 M $ZnSO_4$ + 0.05 $MnSO_4$ electrolytes with and without the presence of C_3N_4QDs.

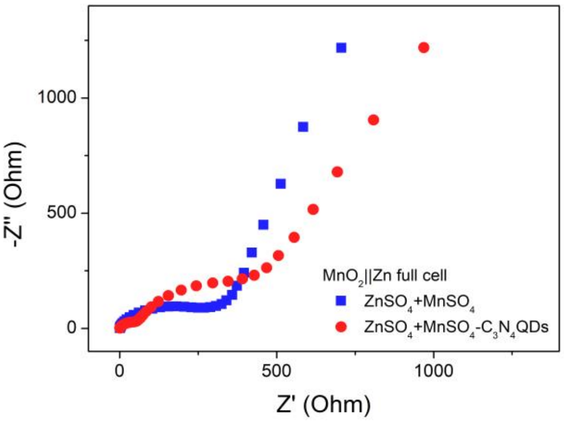

Supplementary Figure 35. Comparisons of EIS spectra for Zn||MnO_2 batteries in 2 M $ZnSO_4$ + 0.05 $MnSO_4$ electrolytes with and without the presence of C_3N_4QDs.

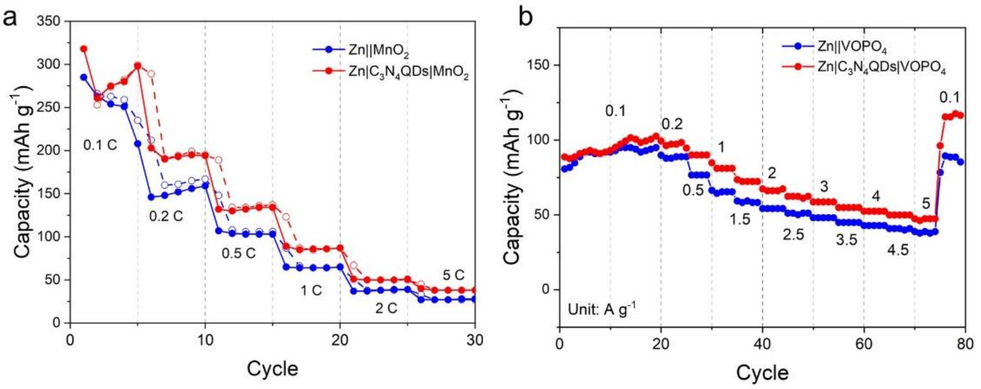

Supplementary Figure 36. (a) Rate performances of Zn|C_3N_4QDs|MnO_2 and Zn||MnO_2 from 0.1 to 5 C, (b) Rate performances of Zn|C_3N_4QDs|$VOPO_4$ and Zn||$VOPO_4$ from 0.1 to 5 A g^{-1}.

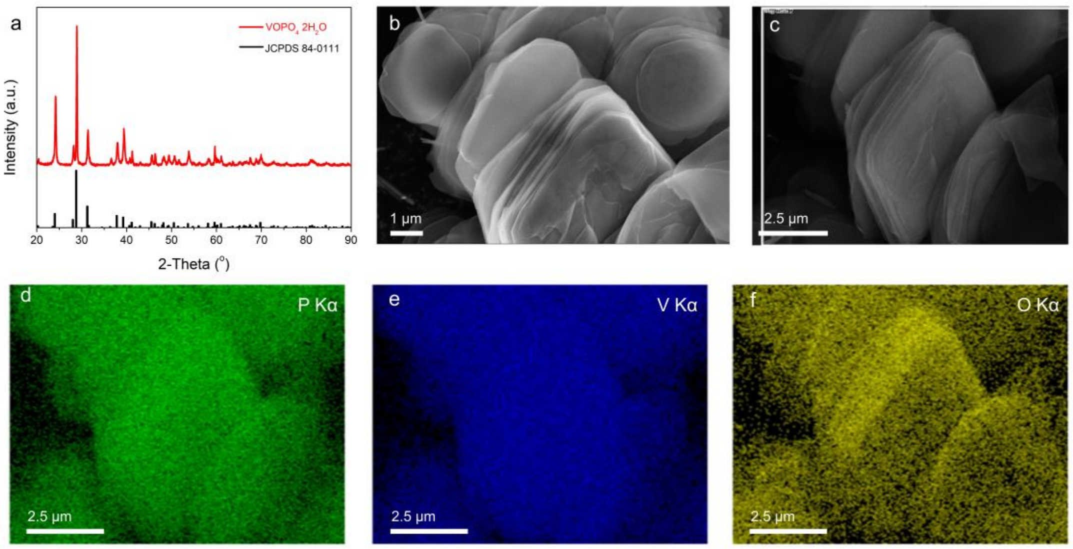

Supplementary Figure 37. Structural and morphology characterization of as-synthesized $VOPO_4$ $2H_2O$. (a) XRD pattern of the synthesized $VOPO_4$ $2H_2O$, (b, c) SEM morphology of the synthesized $VOPO_4$ $2H_2O$, (d – f) Energy dispersive spectroscopy (EDS) mapping of P, V, and O components in the synthesized $VOPO_4$ $2H_2O$.

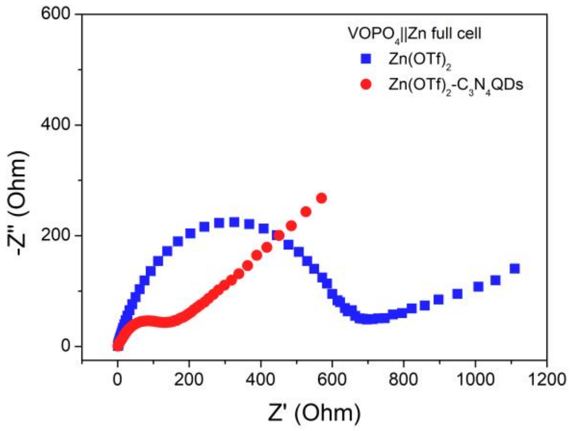

Supplementary Figure 38. Comparisons of EIS spectra for Zn||$VOPO_4$ batteries in 3 M $Zn(OTf)_2$ electrolytes with and without the presence of C_3N_4QDs.

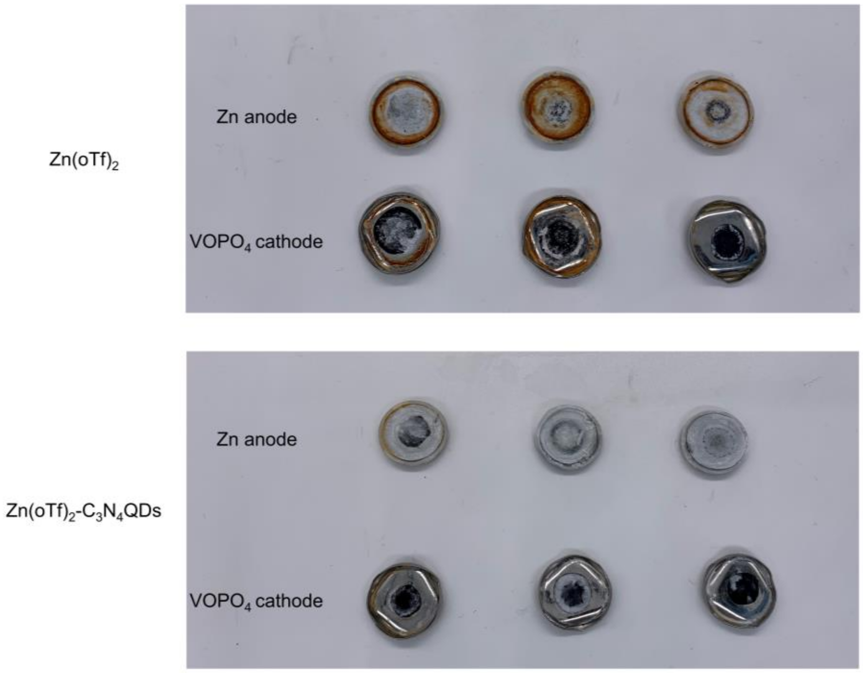

Supplementary Figure 39. Post mortem analysis of the Zn||$VOPO_4$ full cell obtained in 3 M $Zn(OTf)_2$ electrolytes without (top) and with the presence of C_3N_4QDs (bottom).

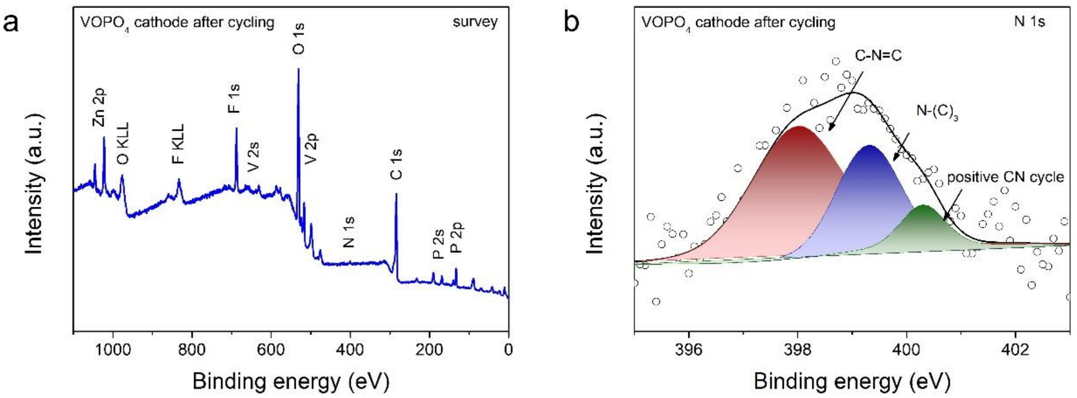

Supplementary Figure 40. (a) XPS survey spectra and (b) high-resolution N 1s spectra of the $VOPO_4$-cathode after the 15th cycle.

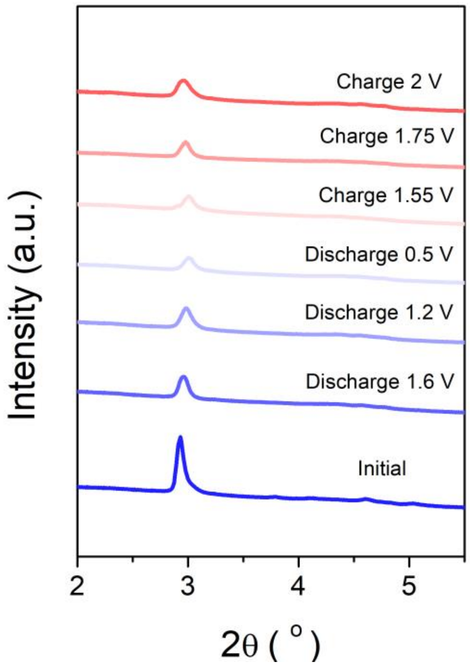

Supplementary Figure 41. The *ex situ* WAXS patterns recorded at different voltage states in Zn||$VOPO_4$ batteries under 3 M $Zn(OTf)_2$ + 0.5 mg mL^{-1} C_3N_4QDs electrolytes.

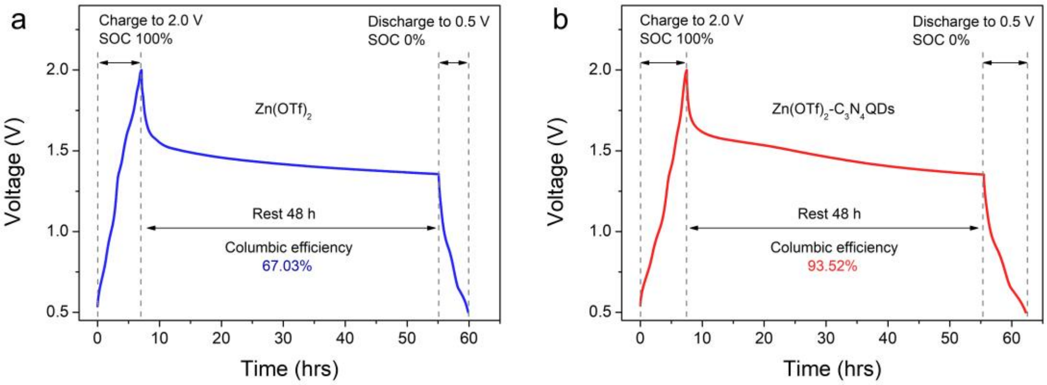

Supplementary Figure 42. Comparisons of open circuit-voltage decay for Zn||$VOPO_4$ batteries in 3 M $Zn(OTf)_2$ electrolytes (a) without and (b) with the presence of C_3N_4QDs. The cells were first fully charged to 2 V at 20 mA g^{-1} and then the cells were rested at 100% stage of charge (SOC) for 48 h, followed by full discharging to 0.5 V.
